# Survived and disappeared intra-oceanic arcs of the Paleo-Asian Ocean: evidence from Kazakhstan

**DOI:** 10.1093/nsr/nwac215

**Published:** 2022-10-11

**Authors:** Inna Safonova, Alina Perfilova

**Affiliations:** LabEPOM, Novosibirsk State University, Novosibirsk 630090, Russia; Igneous Petrology Lab, Sobolev Institute of Geology and Mineralogy, Novosibirsk 630090, Russia; Paleovolcanism and Geodynamics Lab, Zavaritskiy Institute of Geology and Geochemistry, Yekaterinburg 620016, Russia; Faculty of Geosciences and Environmental Engineering, South-West Jiaotong University, Chengdu 610031, China; LabEPOM, Novosibirsk State University, Novosibirsk 630090, Russia; Igneous Petrology Lab, Sobolev Institute of Geology and Mineralogy, Novosibirsk 630090, Russia

**Keywords:** Central Asian Orogenic belt, Paleo-Asian Ocean, Paleozoic, subduction erosion, greywacke sandstones, serpentinite mélange

## Abstract

This paper reviews published and presents new data on U-Pb detrital zircon ages, and petrographic, geochemical and isotope (Sm-Nd, Lu-Hf) compositions obtained from greywacke sandstones of Kazakhstan in order to reconstruct fossil intra-oceanic arcs that once existed at Pacific-type convergent margins of the Paleo-Asian Ocean (PAO) in Paleozoic time. We focus on orogenic belts of central Kazakhstan (Itmurundy and Tekturmas) and eastern Kazakhstan (Zharma and Char) in the western Central Asian Orogenic belt. These orogenic belts host accretionary complexes with greywacke sandstones of early Paleozoic (central Kazakhstan) and middle-late Paleozoic (eastern Kazakhstan) ages. First, we evaluate general perspectives for studying sandstones to reconstruct survived and disappeared magmatic arcs, taking into account episodes of subduction erosion. Then we discuss the analytical data from sandstones to make conclusions about the ages and formation settings of their igneous protoliths and define maximum deposition ages. Finally, we discuss the role of serpentinite mélanges in tectonic reconstructions. We argue that sandstones hosted by accretionary complexes are typically greywackes deposited close to their igneous sources and buried rapidly. The provenances of the studied greywacke sandstones of central and eastern Kazakhstan were dominated by mafic to andesitic igneous protoliths derived from juvenile mantle sources. The igneous rocks in the provenances were emplaced in an intra-oceanic arc setting. The sandstones were deposited in fore-arc/trench basins or, to a lesser degree, in back-arc basins. The data from both sandstones and serpentinite mélanges reconstruct middle-late-Cambrian, Ordovician, late-Devonian and Carboniferous arcs of the western PAO. The middle-late Cambrian arcs were fully destroyed by subduction erosion, whereas the Ordovician and Carboniferous arcs survived. The late-Devonian arcs were also eroded, but partly. Both the early and late Paleozoic active margins of the PAO were characterized by alternating periods of accretionary growth and subduction erosion.

## Teaser

The CAOB is dominated by recycled crust although geologically it represents a Pacific-type orogenic belt. A probabe reason is tectonic erosion of juvenile arcs leaving pieces in serpentinite melange and greywacke sandstones, which thus represent true and unbiased archives of both survived and disappeared fossil intra-oceanic arcs.

## INTRODUCTION

The proportions of juvenile versus recycled crust in intracontinental orogenic belts that formed in place of former paleo-oceans is a key issue of tectonic and metallogenic reconstructions. The suturing and closure of the Paleo-Asian Ocean—an ocean that once existed between the Siberian, North-China, Tarim and Kazakhstan continents—formed the Central Asian Orogenic belt (CAOB), the world’s largest Phanerozoic intra-continental foldbelt. Since the landmark book by Zonenshain *et al.* [1], the CAOB has been studied by dozens of research groups worldwide [2–6]. However, the nature of the crust of the CAOB, juvenile versus recycled, has remained debatable, in particular due to the contradictory geochemical and isotope data obtained from igneous rocks and their hosted zircons [5,7,8].

**Figure 1.**
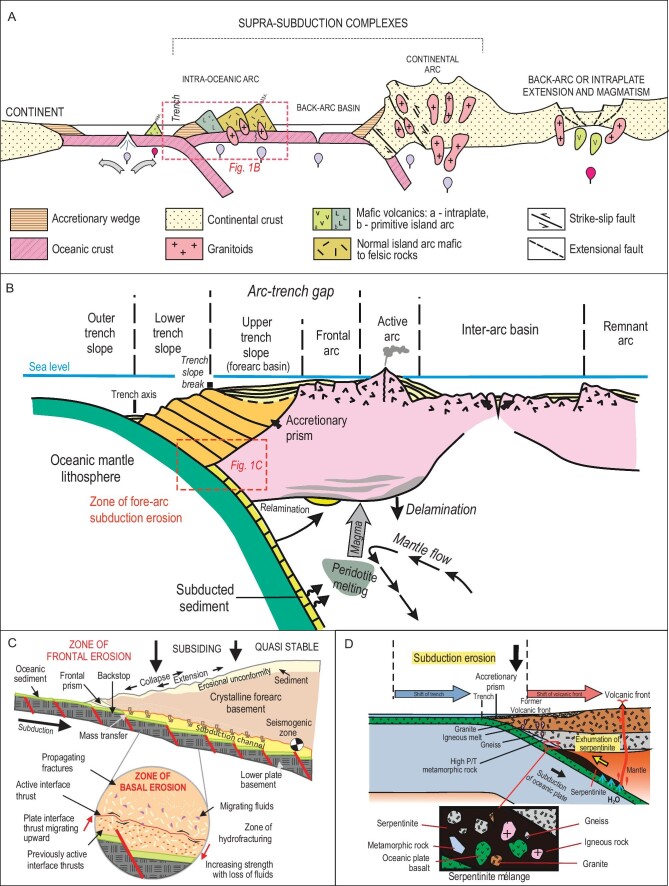
Formation and transformation of continental crust at Pacific-type convergent margins. (A) A profile illustrating formation of new and recycled crust at Pacific-type convergent margins (adapted from Refs [5,16]). (B) A scheme of a Pacific-type convergent margin showing an intra-oceanic subduction system and loci of related magmatism, sedimentary basins and subduction erosion (adapted from Ref. [15]). (C) Mechanisms of subduction erosion through fracturing and transportation of detached fragments into a subduction channel (taken from Ref. [17]). (D) Formation of serpentinite mélange with fragments of tectonically eroded pieces of OPS and arc basement rocks captured beneath the previous volcanic front at a shallower depth (taken from Ref. [13]).

Juvenile continental crust forms through supra-subduction and intra-plate plume-related magmatism, however, the major sites of the growth of juvenile continental crust are intra-oceanic or island arcs (Fig. 1A) [9,10]. Traditionally, island-arc terrains have been reconstructed through study of igneous rocks formed in supra-subduction tectonic settings. However, during the last few decades it has been shown that island-arc igneous complexes can disappear from the geological record because of tectonic erosion [11] (Fig. 1B and C). Geological criteria for identification of episodes of subduction erosion in ancient orogenic belts are: (i) disappearance of peaks of U-Pb detrital zircon ages in arc-derived turbiditic greywacke sandstones of different ages; (ii) small sizes of outcrops of igneous rocks possessing supra-subduction geochemical affinities; (iii) reduced (compared to actualistic analogues) distance between trench and arc; (iv) presence of blocks of mafic to felsic arc-related igneous rocks in serpentinite mélanges; (v) magmatic lull and migration of trench landward [5,12–15].

Erosion of a magmatic arc leaves either clastic rocks, typically greywacke sandstones, often parts of trench/fore-arc/back-arc turbidite associations (Fig. 1B), or blocks in serpentinite mélange (Fig. 1D). Until recently, not much attention has been paid to detailed studies of bulk-rock chemical composition, zircon U-Pb ages and Hf isotopes of such sandstones, in particular those formed in relation to Pacific-type orogeny [12,18]. However, progress in analytical technologies and methods (mass U-Pb dating of detrital zircons and their Lu-Hf systematics) has made greywacke sandstones a key part of the study of fossil intra-oceanic and continental magmatic arcs (Fig. 1A). The revived method of petrographic counting [19,20], the geochemistry of rocks and the isotope signatures of zircons carry important information about the age, petrogenesis and mantle sources of parental igneous rocks in the provenance and about the age of sedimentation [21–23]. Such geochemical and isotope measurements allow us to prove the formation of parental igneous rocks in correlation with regional stages of tectonic evolution. Nowadays, scientific papers on supra-subduction and/or accretionary complexes that do not provide high-precision bulk-rock geochemical and isotope data and U-Pb and Hf zircon data from clastic rocks formed by direct destruction of magmatic arcs, both intra-oceanic and continental, may be lacking critical information.

**Figure 2.**
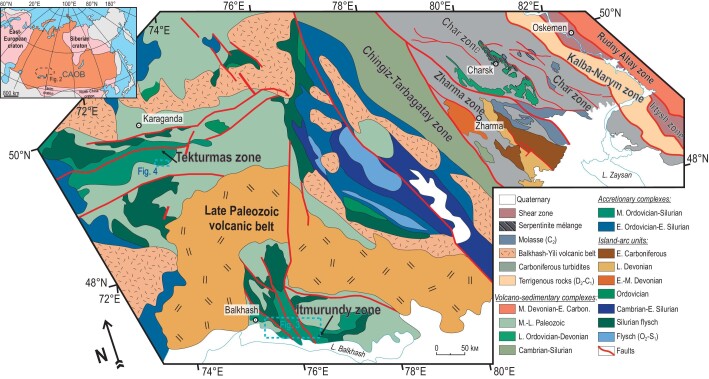
A tectonic map of central and eastern Kazakhstan and adjacent territories showing the location of the Itmurundy, Tekturmas, Zharma and Char zones (adapted from Refs [22,36]).

Recently, more and more papers have been published on the composition, U-Pb zircon ages and Hf-in-zircon isotopes of sandstones from different orogens of the CAOB (Fig. 2): Altai [21,24,25], Tienshan [23,26–28], Mongolia [21,29,30] and Kazakhstan [22,31,32]. However, a lot of supra-subduction and turbiditic formations remain understudied. In this paper we present both new and published data, U-Pb zircon ages, bulk-rock geochemistry, and Sm-Nd and Lu-Hf isotopic ratios, from greywacke sandstones of four orogenic belts in the western CAOB: Itmurundy and Tekturmas (early Paleozoic) in central Kazakhstan and Zharma and Char (middle-late Paleozoic) in eastern Kazakhstan (Fig. 2). All four orogenic belts include accretionary and supra-subduction complexes formed during the Paleozoic evolution of the Paleo-Asian Ocean and its active margins. Special attention will be given to the methods and approaches used for studying sandstones, as well as their potential for reconstructing survived and disappeared arcs. In addition, we will shortly discuss serpentinite mélanges hosting pieces of supra-subduction igneous rocks. Finally, we will discuss survived and disappeared arcs in central and eastern Kazakhstan.

## FROM MAGMATIC ARC TO SANDSTONE

Intra-oceanic arcs (IOAs) producing juvenile continental crust [9] develop over subduction zones, where the lithosphere of one oceanic plate submerges under that of another oceanic plate (Fig. 1A and B). The average volume of new crust generated at IOAs each year is ∼2.5–2.7 km^3^ [33]. However, large volumes of juvenile arc material can be eroded from both the surface and the bottom [11,34]. The surface erosion forms thick greywacke sandstones, often part of turbidite associations, deposited in back-arc and fore-arc basins and in fore-arc trenches (Fig. 1B). In places, magmatic arcs can be fully destroyed during accretion- or collision-related orogenic processes, leaving clastic sediments (turbidite basins) with only minor or no outcrops of magmatic rocks [22,30,35]. Tectonic or subduction erosion can destroy an arc from its bottom/root through the interaction of the hanging wall of subduction zone, which *de facto* is the bottom of the arc, with oceanic floor topography and through the fracturing caused by hydration and bending of the subducting slab (Fig. 1C).

The destruction of magmatic arcs, both intra-oceanic and continental, and the transportation of the eroded material down to the trench, results in the formation of characteristic sedimentary clastic rocks: greywacke sandstones, a typical constituent of turbidite. Unlike arcs, these sandstones commonly remain on the surface, allowing us to determine the nature of a former magmatic arc—intra-oceanic or continental (Fig. 1A). To reconstruct the age, composition and mantle source of protolithic igneous rocks, the destruction of which formed greywacke sandstone, we should study the geological relationships of clastic rocks, and analyze their bulk-rock chemical composition, Nd isotope systematics and Hf-in-zircon isotope ratios.

Of special importance is the U-Pb dating of detrital zircons hosted by sandstones. For example, the first evidence for the disappearance of an Ordovician intra-oceanic arc from the geological record of the Japanese Islands in early Carboniferous time came from the U-Pb age spectra of detrital zircons from turbiditic sandstones of different ages cropped out on Honshu Island [12].

An important issue is how to distinguish back-/fore-arc and trench clastic formations. Turbidite accumulated in a trench is a part of Ocean Plate Stratigraphy or OPS [37]. OPS represents a regular succession of sedimentary and magmatic rocks that, respectively, deposited or erupted on the sea floor as the underlying oceanic plate was traveling from the site of its birth at a mid-ocean ridge to its demise at a deep-sea trench [37,38]. A typical OPS succession includes: (i) pelagic chert often deposited directly onto oceanic basalts (mid-ocean ridge basalt, MORB, or oceanic island basalt, OIB); (ii) hemipelagic siliceous shale, greywacke and mudstone formed closer to a trench and (iii) trench turbidites. OPS turbidites typically occur in the upper parts of OPS packages [22,31]. Fore-arc and back-arc clastic formations contain greywacke sandstones, which can also be parts of banded turbidites or represent massive, not stratified, deposits. However, the sandstones deposited in back-arc basins can contain material derived from both an intra-oceanic arc and adjacent continental arc, i.e. contain coeval young arc-derived zircons and older zircons derived from continental and/or evolved arc basements. In addition, they may exhibit mixed isotopic features, i.e. negative to positive ϵNd(t) and ϵHf(t) [8,39,40]. In general, the greywacke sandstones that deposited in coeval trench, fore-arc and/or back-arc basins can be derived through destruction of their separating magmatic arc (Fig. 1A and B), and therefore their composition should match that of arc magmatic rocks. Thus, turbidites and greywackes available to our study represent an important source of information about their protolithic magmatic arcs.

**Figure 3.**
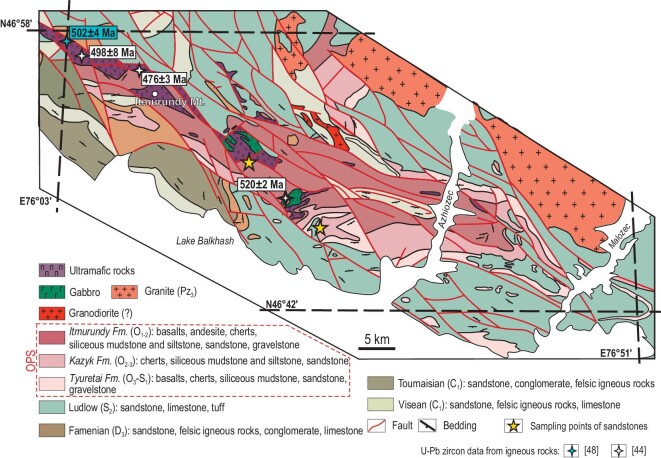
Geological scheme of the Itmurundy zone including a Pacific-type orogenic belt based on the 1/200 000 Geological map of the USSR, Sheet L-43-XI (adapted from Refs [48,49]).

**Figure 4.**
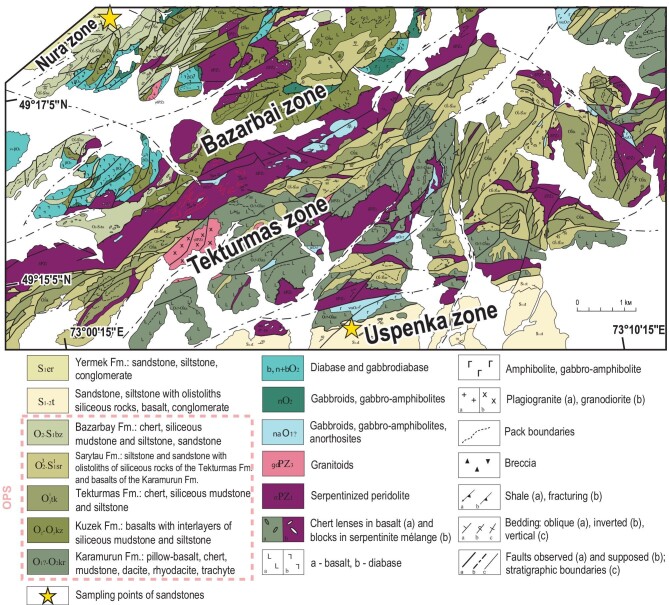
Geological scheme of the Tekturmas belt, central Kazakhstan (adapted from Ref. [55]).

The sandstones derived from an intra-oceanic magmatic arc should have geochemical features that accord well with those typical of intra-oceanic arc igneous series, e.g. tholeiitic and calc-alkaline, mafic to andesitic. Then, the U-Pb ages of the detrital zircons of sandstones must have unimodal distributions suggesting their derivation from a single intra-oceanic arc. Third, their bulk-rock Nd and Hf-in-zircon isotope compositions must be indicative of juvenile sources, i.e. show positive ϵNd(t) and ϵHf(t) values. We suggest a parental continental arc (Fig. 1A) for sandstones if: (i) the distribution of the U-Pb ages of their derived detrital zircons is polymodal, i.e. includes older ages of zircons probably derived from older terrains; (ii) the composition fits sub-alkaline andesitic and felsic rocks (e.g. dacite-granodiorite, rhyolite-granite) and (iii) the values of ϵNd(t) and ϵHf(t) are negative. Below we will illustrate such an approach based on U-Pb ages and petrographic, geochemical and isotope data from OPS sandstones hosted by orogenic belts of central (Itmurundy and Tekturmas) and eastern (Zharma and Char) Kazakhstan (Figs 3 and 4).

## GEOLOGICAL OVERVIEW OF CENTRAL KAZAKHSTAN

Subduction of oceanic crust forms Pacific-type orogenic belts that typically include supra-subduction igneous complexes and accretionary complexes, but not only those (Fig. 1A). There are many Pacific-type orogenic belts in the western CAOB in general, and in Kazakhstan in particular, ranging in ages from Vendian to late Paleozoic [1,4,5,8,36]. The Itmurundy and Tekturmas Pacific-type orogenic belts are major orogenic structures of central Kazakhstan (Fig. 2). Tectonically, they are part of the Junggar-Balkhash folded system in the western CAOB, formed by the suturing of the Junggar-Balkhash Ocean, a western branch of the Paleo-Asian Ocean [4,36,41,42]. The Itmurundy and Tekturmas belts host late Cambrian-Ordovician supra-subduction igneous complexes and Ordovician-to-early-Silurian accretionary complexes, which both, until recently, had remained poorly studied [31,32,43–48]. Both belts are composed of tectonic sheets separated by thrust and strike-slip faults. The igneous and sedimentary rocks often look strongly deformed and/or folded.

### Itmurundy orogenic belt

The Itmurundy orogenic belt is extended along Lake Balkhash over a distance of >80 km and consists of an ophiolitic belt and an accretionary complex (Fig. 3). The middle-late Cambrian ophiolitic association includes ultramafic and mafic rocks (harzburgite, wherlite, dunite, gabbro) and serpentinite mélange hosting fragments of sedimentary (chert), metamorphic (amphibolite) and igneous (plagiogranite, diorite) rocks [31,43,45,48,50]. The accretionary complex includes Ordovician-to-early-Silurian OPS igneous and sedimentary rocks [37]: MORB- and OIB-type basalts, pelagic ribbon chert, hemipelagic siliceous mudstone and siltstone and trench turbidite [47]. The Itmurundy orogenic belt includes lithologies of three formations: Itmurundy (O_1-2_), Kazyk (O_2-3_) and Tyuretai (O_3_–S_1_) [49] (Fig. 3). The Itmurundy Fm. consists of basalt and andesibasalt, ribbon chert, siliceous mudstone and siltstone, and subordinate sandstone. Its early-middle Ordovician age was constrained by conodonts in ribbon chert and siliceous mudstone [44]. The Kazyk Fm. is dominated by red chert containing middle-late-Ordovician conodonts and siliceous mudstone and siltstone, plus subordinate igneous (basalt) and clastic (sandstone) rocks [44,47,51]. The Tyuretai Fm. consists of thick fine-to-coarse-grained sandstone and gravelstone, siliceous siltstone, siliceous mudstone, chert and basalt. The late Ordovician–late Silurian age of the sandstones was determined by U-Pb detrital zircon ages [31], conodonts in chert [44,51] and graptolites in siltstone [52].

### Tekturmas orogenic belt

The 200 km long Tekturmas orogenic belt is located south of Karaganda (Fig. 2) and consists of four zones (from NW to SE): Nura, Bazarbai, Tekturmas and Uspenka (Fig. 4). The major associations are ophiolites, accretionary complex units and clastic deposits. The ophiolitic association of the Tekturmas zone includes ultramafic-mafic rocks, plagiogranite and serpentinite mélange with fragments of granitoids, basalt and OPS sediments [43,45,46]. The accretionary complex also includes OPS units cropping out within the Tekturmas and Bazarbai zones. In the Tekturmas zone, the OPS units are attributed to the Karamurun (O_1?-2_kr), Tekturmas (O^1^_3_tk) and Sarytau (O^2^_3_–S^1^_1_sr) formations (Fig. 4). The Karamurun Fm. consists of sheared OIB-type pillow-lavas [46] with lenses and interbeds of chert and siliceous mudstone. Its middle-Ordovician age (middle Darriwilian) was determined by conodonts in chert [51]. The Tekturmas Fm. is dominated by chert and siliceous mudstone with subordinate siliceous siltstone. Both chert and siliceous mudstone often have a ribbon texture and look strongly folded/duplexed. The middle-late-Ordovician age of the formation was constrained by conodonts in chert [53]. The Sarytau Fm. (early Silurian) represents olistostrome consisting of siliceous siltstone and sandstone and olistoliths of siliceous sediments and basalts. The matrix of the olistostrome contains late-Ordovician conodonts and early Silurian graptolites [54].

In the Bazarbai zone, the OPS units belong to the Kuzek (O_2-3_kz) and Bazarbai (O_2_-S_1_bz) formations. The Kuzek Fm. is similar to the Karamurun Fm. as it is predominantly basaltic with subordinate chert, siliceous mudstone, andesibasalt and plagiogranite. However, the Kuzek basalts possess supra-subduction geochemical characteristics [46]. The siliceous mudstones contain upper-Darriwilian (middle-Ordovician) and Sandbian (upper-Ordovician) conodonts [43]. The U-Pb zircon age of the plagiogranite is 489 ± 8 Ma [43]. The Bazarbai Fm. is lithologically similar to the Tekturmas Fm. as it is dominated by ribbon chert with thin interbeds of siliceous mudstone and siltstone, and tuffs. The middle-upper Ordovician age of the formation is constrained by conodonts in chert and siliceous siltstone [53]. The Nura and Uspenka zones are dominated by clastic rocks of the Yermek Fm. and Sarytau Fm. [46], respectively. We consider here the greywacke sandstones of the Nura and Uspenka zones [32] (Fig. 4).

## GEOLOGICAL OVERVIEW OF EASTERN KAZAKHSTAN

The Zharma and Char orogenic belts are located in eastern Kazakhstan, east of Zharma and west of Oskemen cities (Fig. 2). Tectonically they both belong to the Ob′-Zaisan folded system formed by the evolution and suturing of the Ob′-Zaisan branch of the Paleo-Asian Ocean [3]. The Ob′-Zaisan folded system hosts late-Devonian to early Carboniferous igneous complexes and sedimentary formations formed at active margins of the Kazakhstan and Siberian continents.

The Zharma Devonian-early Carboniferous island-arc terrane or Zharma OB is separated from the early Paleozoic Chingiz-Tarbagatay island-arc terrane in the southwest and the late Paleozoic Char orogenic belt (Char OB) in the northeast (Fig. 2). The Devonian mafic to felsic igneous rocks and sandstones are unconformably overlain by early Carboniferous terrigenous rocks (sandstone, siliceous mudstone and siltstone), mafic volcanic rocks and limestones. The main constituents of the Zharma OB are Givetian-Fransian (D_2_zv-D_3_f) and Famennian (D_3_fm) volcanogenic-sedimentary units, and Kokon’ (_1_kk) and Koyandin (C_?_kn) formations. The Givetian-Fransian unit consists of siltstone, polymictic sandstones, gravelstone and volcanic rocks, andesite, andesibasalt and basalt [10,22]. The Famennian unit includes chert, siliceous siltstone, clay shale, polymictic sandstones, andesitic lavas and tuffs. The Kokon’ and Koyandin formation are similar in age, but the Kokon’ Fm. consists of terrigenous rocks (siliceous siltstone, shale, sandstones), whereas the Koyandin Fm. is dominated by basalt, andesibasalt, andesitic lava and tuff with subordinate siliceous shales, siltstone and sandstones. Greywacke sandstones occur in all units and formations [22,56].

The NW-SE trending Char OB represents an axial suture structure of the Ob′-Zaisan folded system and is extended over a distance of >300 km at a width of 7–10 km only. It borders the middle-Paleozoic Rudny Altai terrane in the northeast and Zharma OB in the southwest (Fig. 2). The Char OB possesses a very complicated structure including oceanic and supra-subduction ophiolites, HP-LT metamorphic rocks and OPS units [3,57,58]. The ophiolitic association includes serpentinite mélange with blocks of ca. 323 Ma tonalite and plagiogranite [59] and separate small bodies of Devonian-to-early-Carboniferous supra-subduction volcanic and subvolcanic rocks [58]. The accretionary complex includes a complete succession of OPS rocks: MORB-type and OIB-type basalt, ribbon chert, siliceous mudstone, siltstone, shale and sandstone [57]. Greywacke sandstones occur in the upper parts of OPS sections [22]. The Devonian-to-early-Carboniferous age of the sediments was constrained by late-Devonian radiolarians and Tournaisian conodonts in cherts of the Karabaev Fm., Famennian conodonts in carbonates of the Urumbai Fm., Tournaisian radiolarians and conodonts in chert and siliceous mudstone of the Verochar Fm. [60,61]. The younger clastic Dalankarin and Taubin formations consist of fine-grained siliceous sediments and sandstones containing Bashkirian and Moscovian conodonts, respectively [58]. The sandstones of the Char OB studied in reference [22] belong to the Dalankarin and Toubin formations.

## U-Pb AGES OF DETRITAL ZIRCONS FROM SANDSTONES

**Figure 5.**
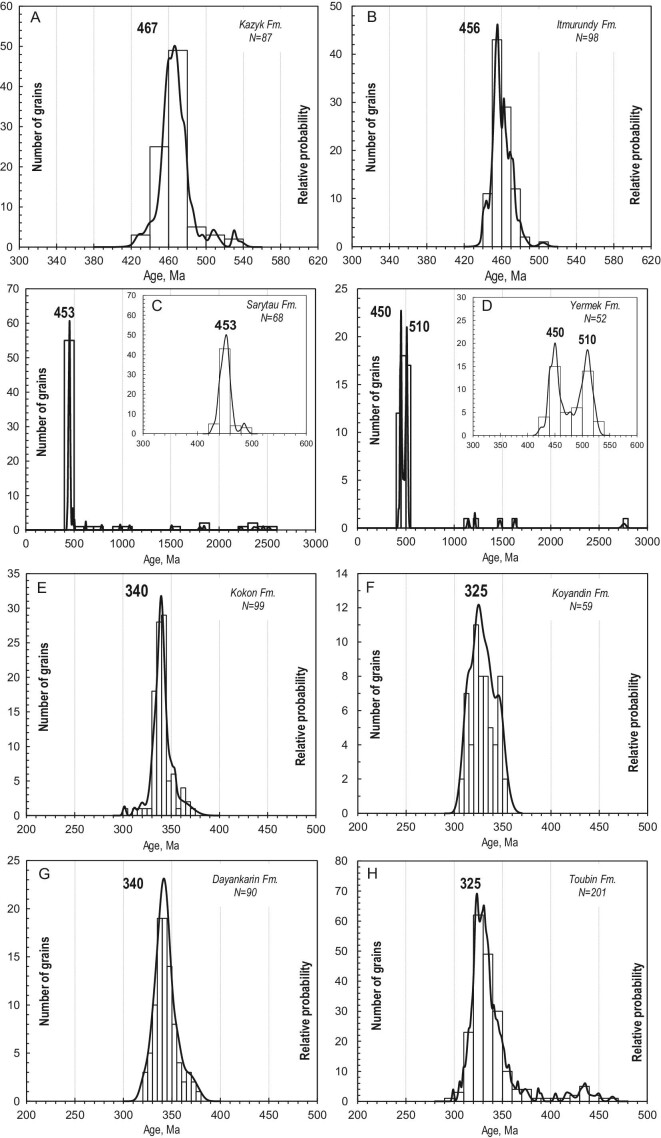
Distributions of U-Pb ages of detrital zircons from greywacke sandstones of the (A and B) Itmurundy, (C and D) Tekturmas, (E and F) Zharma and (G and H) Char belts (originally published in Refs [22,31,32,56]).

We obtained a ∼760 U-Pb detrital zircon age of 100%–95% concordance from greywacke sandstones of four orogenic belts [22,31,32,56] (Supplementary Table S1). The color, space and size of zircons are similar. The zircon grains are 30 to 200 μm long, clear or light yellow, and euhedral (stubby to prismatic) to subhedral (slightly round or barrel-shaped) in shape suggesting relatively short transportation before deposition. All zircons are characterized by oscillatory zoning seen in CL images and have Th/U values higher than 0.1 but less than 2, implying their igneous origin [62]. Figure 5 shows that the distribution patterns of U-Pb zircon ages from greywacke sandstones of all four belts are unimodal, suggesting their derivation from an intra-oceanic arc [22] (Fig. 1B).

### Itmurundy OB

About 200 zircons from the sandstones of the Itmurundy and Kazyk formations yielded U-Pb age distributions peaked at 456 and 476 Ma, with the youngest clusters at 439.0 ± 7 and 442.0 ± 3 Ma, respectively (Fig. 5A and B), suggesting an early-to-middle-Ordovician intra-oceanic arc [31]. There are also several ages at 512–480 Ma. Thus, the U-Pb zircon ages suggest two magmatic arcs in the provenance: the Cambrian arc, which was destroyed by subduction erosion and survived only as blocks in mélange, and the Ordovician arc, which survived as igneous complexes and as derived greywacke sandstones [31,44,47].

### Tekturmas OB

About 140 zircons from two sandstones of the Yermek and Sarytau fms. yielded U-Pb age distributions peaked at 453 and 450 Ma (late Ordovician), with the youngest clusters at 437 ± 7 and 438 ± 9 Ma, respectively [32] (Fig. 5C and D). Of special interest is the second peak in the histogram of the Nura sample at 510 Ma (Fig. 5C). Both Tekturmas sandstones record a late-Ordovician intra-oceanic arc [32] (Fig. 5C and D). That arc has probably survived as the mafic to andesitic volcanic rocks of the Bazarbai zone (Fig. 4). However, no isotopic ages have been obtained so far from coherent (not in mélange) igneous rocks of the Kuzek or Karamurun formations.

### Zharma OB

About 160 zircons from the sandstones of the Kokon’ and Koyandin (?) fms. yielded unimodal U-Pb age distributions peaked at 340 and 327 Ma, respectively (Fig. 5E and F), suggesting Visean and Serpukhovian intra-oceanic arcs as their respective sources [22,56]. The youngest clusters at 319 ± 3 and 312 ± 3 Ma indicate the Bashkirian and Moscovian maximum deposition ages of the Kokon’ and Koyandin formations, respectively. The oldest ages of zircons from the Kokon’ Fm. (Visean arc) form a small late-Devonian peak (371–360 Ma) (Fig. 5E). The age distribution of zircons from the Koyandin Fm. (Serpukhovian arc) shows a smaller Visean peak at ca. 340 Ma, i.e. the provenance of the Koyandin sandstones included the same igneous rocks, which delivered zircons to the Kokon’ sandstones. The oldest ages for the Serpukhovian arc are all in the Tournaisian (354–350 Ma), forming no separate peaks (Fig. 5F). Thus, the Zharma sandstones keep records of three arcs: late Devonian, Tournaisian-Visean and Serpukhovian.

### Char OB

About 250 zircons from the sandstones of the Dalankarin and Toubin formations yielded peaks of U-Pb detrital zircon ages at 340 and 325 Ma, respectively [22,56] (Fig. 5G and H). The youngest clusters at 321 ± 5 and 311 ± 2 Ma indicate the respective maximum deposition age in the Bashkirian and Moscovian, respectively. The data from the Char OB also suggest Visean and Serpukhovian intra-oceanic arcs. Similar to the Zharma OB, the oldest ages of zircons from the Bashkirian sandstones (373–369 Ma) indicate a late-Devonian arc (Fig. 5G). Probably, the sandstones of the Zharma and Char OBs could be derived from erosion of the same arcs. The oldest ages of zircons from Moscovian sandstones span the interval from 450 to 420 Ma (Fig. 5H), which is the age of an older group of Char supra-subduction igneous rocks having juvenile bulk-rock Nd isotope characteristics (ϵNd(t) = 3.8–7.8) [58]. We suggest three scenarios for the occurrence of Ordovician to Silurian zircons in the Moscovian sandstones. (i) They could come from the late-Ordovician-early-Silurian arc, pieces of which have been preserved in the Char OB. (ii) They are xenogenic, i.e. were captured by the parental magma during its ascent. (iii) Their provenance was different from that of the Char Bashkirian sandstones (Fig. 5E and F) and from that of the Zharma Moscovian sandstones (Fig. 5F and H).

## PETROGRAPHY, GEOCHEMISTRY AND ISOTOPES OF GREYWACKE/TURBIDITE SANDSTONES

### Petrography

**Figure 6.**
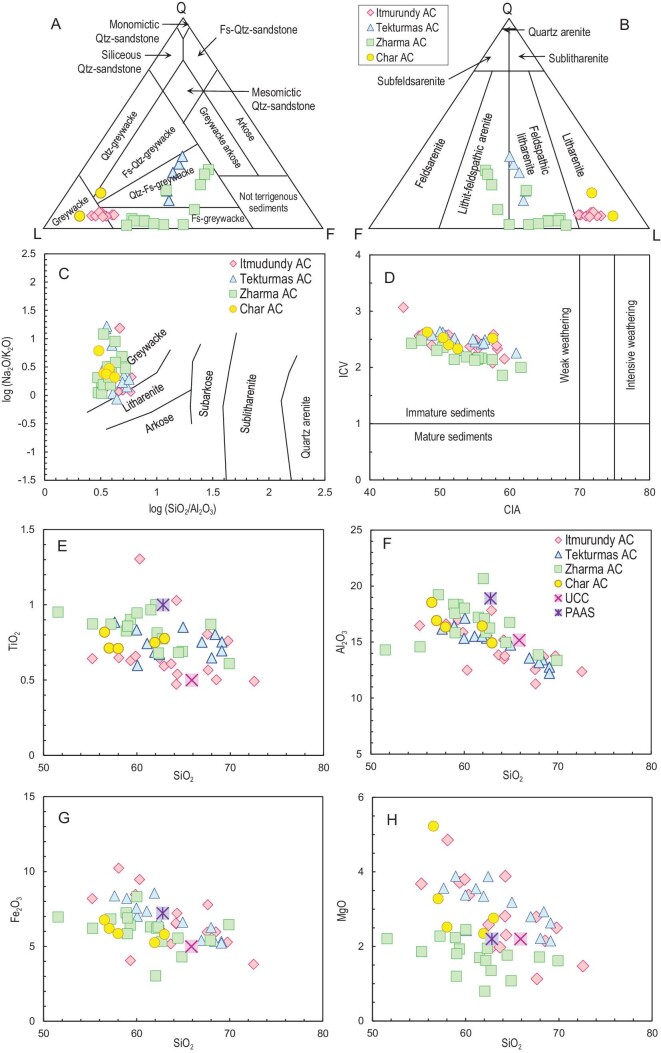
Petrographic and geochemical characteristics of greywacke sandstones from accretionary complexes of central and eastern Kazakhstan. Classification plots: (A) source: Ref. [19]; (B) source: Ref. [20]; (C) source: Ref. [63]. Geochemical plots: (D) CIA vs. ICV diagram for post-depositional alteration (sources: Refs [64,65]); (E–H) binary SiO_2_ vs. major oxides plots.

The poorly sorted sandstones from all localities are grey to dark-grey and greenish grey. The size of their clasts is variable, from fine grained to coarse grained. The lithic clasts are angular, poorly rounded to half rounded and represented by mafic to andesitic volcanic rocks (20%–50%) and sedimentary rocks (10%–30%). The volcanic rocks typically have aphyric, hyalopylitic and porphyric structures and are dominated by plagioclase laths and dark mesostasis, probably volcanic glass, often chloritized. The sedimentary rocks are commonly chert, siliceous siltstone and mudstone. The mineral grains are feldspar (5%–20%) and quartz, monocrystalline and polycrystalline (6%–8%). The feldspar grains are mostly plagioclase, however, a limited amount of K-feldspar grains is also present. Plagioclase fragments and laths are often saussuritized. There are also accessory zircon, titanite, mica (muscovite and biotite) and opaque minerals. The secondary minerals are epidote, chlorite, Fe-hydroxides and calcite. Most samples show no cement, only fine-grained silty matrix, the portion of which is <10%. According to the classifications of [19,20] based on petrographic counting, the majority of the sandstones are greywackes and litharenites (Fig. 6A and B) (Supplementary Table S2). For more details of the petrographic compositions and for microphotos, see [22,31,32].

### Bulk-rock geochemistry

The bulk-rock geochemical features of the greywacke sandstones from all four regions are surprisingly similar [22,31,32]. The contents of most major oxides are variable: SiO_2_ = 50.1–69.8, TiO_2_ = 0.5–1.3, Al_2_O_3_ = 8.8–20.7, Fe_2_O_3_ = 3.0–13.4, MgO = 1.0–6.5, Na_2_O = 2.5–7.4, K_2_O = 0.1–3.9 wt% (Supplementary Table S3). All sandstones are characterized by decreased Al_2_O_3_ and increased Fe_2_O_3_ and MgO contents relative to post Archean Australian shale (PAAS) suggesting the presence of compositionally mafic to andesitic rocks in the provenances. More evidence for the greywacke nature of the sandstones comes from the geochemistry-based classification [63], also showing that most samples are greywackes, as in (Fig. 6C). The chemical index of alteration (CIA) [64] characterizes strong (CIA > 70) to weak (CIA < 70) weathering. The index of chemical variability (ICV) [65] characterizes mature (ICV < 1), i.e. rich in clay minerals, and immature (ICV > 1), i.e. rich in igneous minerals (feldspar, pyroxene, amphibole), sediments. The greywacke sandstones under study yielded CIA values ranging from 41 to 58 and ICV values ranging from 1.9 to 3.1, suggesting a low degree of weathering and the immature character of the clastic sediments, respectively (Fig. 6D). The negative trends seen in the binary diagrams for SiO_2_ vs. major oxides with respect to TiO_2_, Al_2_O_3_, MgO and Fe_2_O_3_ (Fig. 6E–H) are typical of supra-subduction igneous series [66] and are similar to those recorded in the volcanic rocks possessing supra-subduction geochemical characteristics from the Itmurundy and Char belts [48,58].

**Figure 7.**
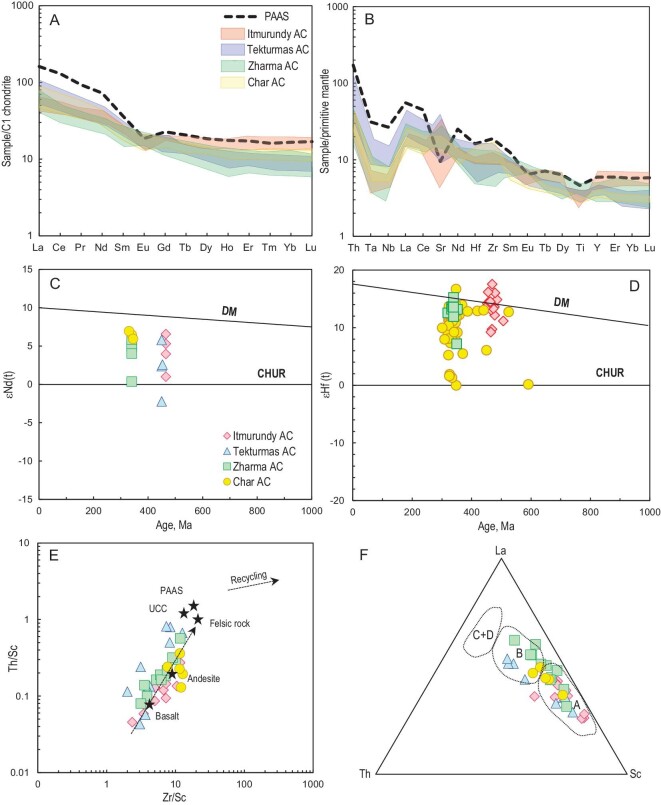
Trace element and isotope characteristics. (A) Chondrite-normalized rare-earth element plots. (B) Primitive mantle-normalized trace element plots. Normalization values are from Ref. [67]. Isotope plots: (C) bulk-rock Nd isotopic compositions; (D) Hf-in-zircon isotopic compositions. Discrimination diagrams: (E) Zr/Sc vs. Th/Sc (source: Ref. [68]); (F) Th-La-Sc (source: Ref. [69]).

In general, the greywacke sandstones from all four regions show relatively short intervals of the concentrations of La (11.8–23 ppm), Nd (12–26.3 ppm), Yb (1.4–2.7 ppm) and total rare-earth elements (REE) (81–165 ppm), and wide intervals of Cr (10–400) and Nb (0.2–70). All the concentrations are below those of PAAS (Supplementary Table S3). All sandstones display similar chondrite-normalized spectra of the concentrations of REE (Fig. 7A). The spectra are moderately to strongly enriched in light REE (LREE) (La_N_/Yb_N_ = 2.4–11.8), slightly down at Eu (Eu/Eu*_av._ = 0.9) and weakly differentiated at heavy REE (HREE) (Gd_N_/Yb_N_ = 1.1–2.1). All spidergrams are characterized by troughs at Nb relative La and Th with Nb/La_pm_ = 0.22–0.56 and Nb/Th_pm_ = 0.12–0.28 (Fig. 7B). Compared to PAAS, most sandstones have lower Th and Cr, probably due to derivation from mafic to andesitic igneous rocks. Part of the samples has high concentrations of Sr, which form clear peaks in the spidergrams, probably as a result of submarine eruptions. Both the REE spectra and spidergrams are similar to those obtained from supra-subduction igneous rocks of respective belts [48,57].

### Bulk-rock Nd and Hf-in-zircon isotopes

The juvenile vs. recycled origin of parental magmatic rocks of greywacke sandstones can be reconstructed by Nd isotopes measured in bulk-rock samples and by Hf isotopes measured in zircons. In our previous papers, we presented the first data on Sm-Nd and Lu-Hf isotope systematics of greywacke sandstones of Kazakhstan [22,31]. In this paper we present several new Nd isotope results from the Zharma zone and the first Nd isotope data from the Tekturmas zone (Supplementary Table S4). For Nd isotope studies we chose the freshest samples without secondary minerals [22,31]. In total, we analyzed Nd isotopes in 15 bulk-rock samples of sandstones, which, according to petrographic and geochemical data (Fig. 6), were formed by direct destruction of intra-oceanic arcs and deposited in fore-arc settings [22,32,33]. The samples have ^147^Sm/^144^Nd = 0.1117–0.1510 and ^143^Nd/^144^Nd = 0.512467–0.512824. Initial isotopic ratios were calculated using microfossil ages and the maximum depositional ages obtained from U-Pb detrital zircon ages [22,31,45,47] (Fig. 5). The isotope diagram shows ϵNd(t) values vs. age (Fig. 7C). All sandstones are characterized by the positive values of ϵNd(t) ranging from 1.0 to 6.9 and the values of T_DM2_ varying from 522 to 709 Ma.

Isotopic ratios of Lu and Hf in zircons were measured in zircons from sandstones of the Itmurundy, Zharma and Char zones (Fig. 7D; Supplementary Table S5). In total, we analyzed the Lu-Hf isotope in ∼100 zircons [22,31,56]. All zircons yielded positive ϵHf(t) values. Seventeen zircons from Itmurundy samples yielded ϵHf(t) values ranging from +9.2 to +17.6 (13.7 in average), with T_DM2_ varying between 854 and 325 Ma. The values of ϵHf(t) in 38 zircons from Zharma sandstones vary from +7.2 to +18.5 (13.6 in average), with T_DM2_ between 322 and 896 Ma. The zircons from Char sandstones yielded ϵHf(t) values varying in a wider interval, from 0 to 16.7. They form two clusters between 0 and 1.9 and between +5.2 and +16.7, with T_DM2_ values ranging from 1350 to 1211 and from 998 to 452 Ma, respectively.

## PROVENANCES AND TECTONIC SETTINGS AS INFERRED FROM GREYWACKE SANDSTONES

All geochronological, petrographic and geochemical data indicate derivation of the sandstones from Kazakhstan accretionary complexes through destruction of igneous rocks. The unimodal character of the distributions of U-Pb detrital zircon ages (Fig. 5) suggests transportation of zircons from a single magmatic arc, and deposition of clastic sediments (turbidites) in fore-arc and trench settings. The most probable sources of those sandstones are intra-oceanic arcs formed over an immature mafic basement, e.g. the modern Mariana arc and Alaska arcs [70,71] or older arcs reconstructed from fore-arc sandstones [72,73]. Turbidites accumulated in a back-arc basin can be contaminated by older zircons from a more felsic and mature continental arc (Fig. 1A). Greywacke sandstones derived from continental arcs are typically characterized by polymodal distributions of U-Pb detrital zircon ages, e.g. the Franciscan accretionary complex, California and Chinese Altai [74–76].

In terms of petrography, the clasts are predominantly lithic and feldspatic (Fig. 6A and B), suggesting fast burial, as lithic fragments and most igneous minerals (pyroxene, feldspar) are less stable during transportation compared to quartz. The bulk-rock compositional features show that the sandstones are greywackes or litharenites (Fig. 6C). Their low CIA and high ICV values (Fig. 6D) indicate their immature character and rapid burial. The trends observed in the binary SiO_2_ vs. major oxides plots suggest the supra-subduction character of the igneous protoliths of the sandstones (Fig. 6E–H). The REE patterns and spider diagrams are also similar to those of subduction-related igneous series [48,58,77], as they are characterized by Eu (REE) and Nb and Ti (spider) negative anomalies (Fig. 7A and B).

The ϵNd(t) (bulk-rock) and ϵHf(t) (zircon) values are positive for most sandstones (Fig. 7C and D), indicating that juvenile mantle sources dominated during the petrogenesis of their igneous protoliths. Such juvenile sources are typical of intra-oceanic subduction systems, at which the contamination of juvenile mantle wedge derived igneous melts by recycled crust material is zero to negligible (Fig. 1). In contrast, sandstones derived from continental arcs or continental basins [78] show negative ϵNd(t), e.g. those of the Chinese Altai and north-eastern Pacific [74,79]. The zero values of ϵHf(t) in two Char zircons and the slightly negative values of ϵNd(t) in one Tekturmas and one Zharma sample may indicate participation of a limited amount of recycled material in igneous petrogenesis and/or contamination of sandstones by clastic material derived from a continental arc and their deposition in a back-arc basin (Figs 1A and B, 7C and D).

In the Zr/Sc vs. Th/Sc discrimination diagram [68], the sandstones of the Itmurundy, Tekturmas and Zharma zones plot between the compositions of basalt and andesite, and those of the Char zone plot close to the composition of andesite (Fig. 7E). Several samples from Tekturmas and Zharma plot closer to dacite. Such strong inter-element correlations (Fig. 7E) also indicate that all greywacke sandstones from the accretionary complexes of Kazakhstan are first cycle sediments and that their provenances were controlled by the composition of igneous protolith rather than by sediment recycling [68]. In the La-Th-Sc discrimination triangle [69], most sandstones plot in the field of intra-oceanic arcs, although several samples of the Tekturmas, Zharma and Char zones plot in the field of continental arc (Fig. 7F). However, as far as most samples are characterized by positive ϵNd(t) (bulk-rock) and ϵHf(t) (zircon) values, we suggest that a part of the Tekturmas, Zharma and Char sandstones were deposited in back-arc basins, which were fed by clastic material supplied by both intra-oceanic arc and continental arc/margin (Fig. 1A). Thus, all petrographic, geochemical and isotope characteristics, coupled with the textural and compositional immaturity of the sandstones, indicate that (i) they accumulated not far from sediment sources and were rapidly buried; (ii) their provenances were dominated by weakly weathered mafic to andesitic igneous rocks; (iii) the parental melts of the igneous protoliths of most sandstones were derived from juvenile mantle sources; (iv) the igneous rocks of the provenances were erupted and/or emplaced in an intra-oceanic arc setting; (v) sandstones were deposited in fore-arc/trench basins or, to a lesser degree, in back-arc basins.

## EVIDENCE FOR DISAPPEARED AND SURVIVED ARCS

### Distribution of U-Pb detrital zircon ages

The distributions of the U-Pb detrital zircon ages obtained from the greywacke sandstones of all four localities provide evidence for partial or complete disappearance of several magmatic arcs. The U-Pb age patterns for Itmurundy sandstones (Fig. 5A and B), peaked at 467 and 456 Ma, indicate an Ordovician long-lived intra-oceanic arc [31]. Note that there are supra-subduction igneous rocks in the Itmurundy zone but their age remains unclear, but supra-subduction ophiolites of that age occur in the adjacent West Junggar region of NW China [80]. There are also 512–480 Ma zircons possessing juvenile Hf isotope characteristics, i.e. positive ϵHf(t) (Fig. 7D). All these data allowed us to suggest two arcs: middle-late Cambrian and Ordovician, which were both destroyed by subduction erosion and for which records have been preserved only in blocks of serpentinite mélange and in greywacke sandstones, respectively.

The U-Pb zircon age distributions obtained from Tekturmas sandstones (Fig. 5C and D) both show peaks at ca. 450 Ma, but only the sandstone of the Sarytau Fm. (Uspenka zone) also shows a peak at 510 Ma (Fig. 5D). The main 450 Ma peak matches the age of supra-subduction granitoids of the Tekturmas zone [46], but no igneous complexes of Cambrian age have been found there so far. The middle-late Cambrian peak detected in the Tekturmas sample (Fig. 5D) fits the 512–480 Ma U-Pb detrital zircon ages detected in Itmurundy sandstones (Fig. 5A and B). Therefore, an extended (Itmurundy to Tekturmas) middle-to-late-Cambrian magmatic arc existed in the Junggar-Balkhash Ocean [42], but later was destroyed by subduction erosion.

The detrital zircon records from the Zharma and Char sandstones are similar as they are both peaked at 340 and 325 Ma (Fig. 5E and H), implying two arcs of early and late Carboniferous ages. However, the Zharma younger sandstone shows both peaks, i.e. 340 and 325 Ma (Fig. 5F), whereas the Char younger sandstone shows no peak at 340 Ma, suggesting partial erosion of the 340 Ma arc in the Char zone. The U-Pb ages of zircons from Char sandstones match those from Char igneous rocks, although there are also late-Devonian igneous rocks in the Char OB [58]. Evidence for a late-Devonian arc also comes from the U-Pb detrital zircon ages of the Zharma OB (Fig. 5E). That arc was probably also destroyed by subduction erosion in Carboniferous time. The U-Pb zircon ages from the southern Zharma OB in NW China [81] match both the late-Devonian and all Carboniferous ages of detrital zircons (Fig. 5E and F). Therefore, both arcs have survived in the Zharma OB, although the Carboniferous arc(s) has been better preserved in both the northern (Kazakhstan) and southern (NW China) parts of the belt compared to the late-Devonian one.

### Serpentinite mélange and subduction erosion

**Figure 8.**
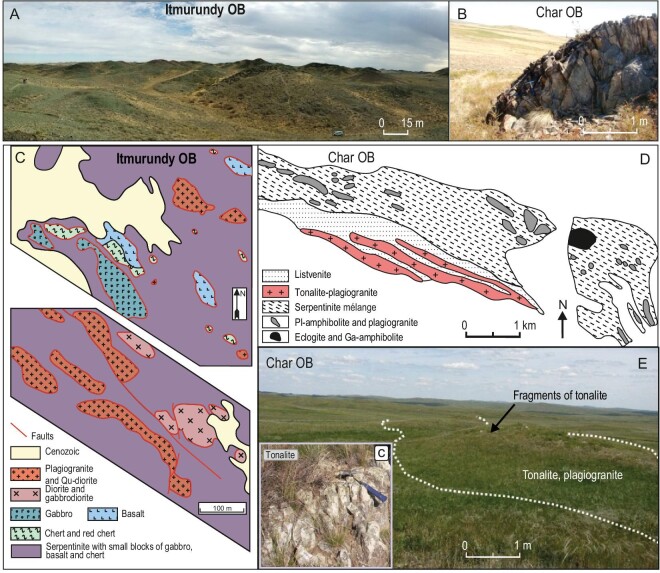
Examples of serpentinite mélanges with fragments of various rocks indicative of subduction erosion. (A and B) Field photos of outcrops of supra-subduction rocks in the Itmurundy and Char belts, respectively. (C) Geological schemes of serpentinite mélanges near Itmurundy Mt. (Fig. 3) reproduced from Ref. [44]. (D and E) Serpentinite mélange with fragments of tonalite reproduced from Ref. [59].

**Figure 9.**
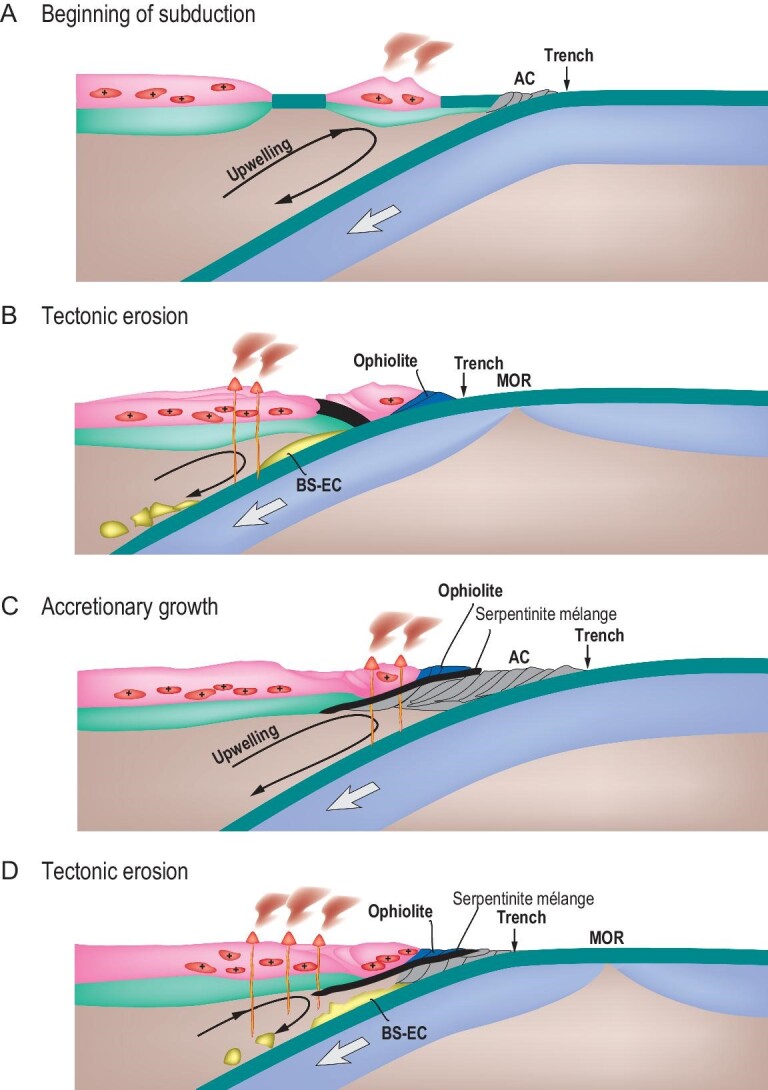
Cartoons illustrating the evolution of an accreting to eroding Pacific-type convergent margin. (A) Beginning of subduction and juvenile magmatism. (B) Tectonic erosion, juvenile magmatism. (C) Accretionary growth oceanward and recycled magmatism. (D) Tectonic erosion, juvenile magmatism (adapted from Ref. [13]).

All localities under consideration, and other orogens of the CAOB like those in western Junggar, Tienshan and northern Mongolia [23,31,42,80,82], are characterized by small coherent, i.e. not in mélange, outcrops of supra-subduction igneous rocks. The sizes of coherent outcrops of supra-subduction rocks are several meters to several dozen meters long/wide only (Fig. 8A and B), which is almost negligible compared to the visible on-land part of magmatic arcs, which may reach 3000–4000 m, e.g. the Fuji Mt. in Japan or the Andes in South America. Supra-subduction igneous rocks often occur as fragments in serpentinite mélange (Fig. 8C and D). Serpentinite mélange, also referred to as ophiolitic mélange, is a typical constituent of Pacific-type convergent margins and is emplaced between subduction and accretionary complexes (Fig. 1D). The fragments of different lithologies can be detached from the arc basement and subducting oceanic slab, and get captured by serpentinite during destruction of the bottom of the subduction zone hanging wall and serpentinite exhumation [13] (Figs 1D and 9).

The Itmurundy, Tekturmas and Char OBs host serpentinite mélanges with arc granitoids (Figs 2–4, 8C–E), whose ages are either slightly greater than the age of arc magmatism as recorded in U-Pb detrital zircon ages (Fig. 5), or similar [31,44,45,48,59]. The 512 to 480 Ma U-Pb ages of zircons from Itmurundy sandstones (Fig. 5A, B and D) accord well with the U-Pb zircon ages of Itmurundy supra-subduction granitoids in mélange [44,48], but differ from the 473 and 453 Ma Tekturmas plagiogranites also present as fragments in serpentinite mélange [36,45] (Figs 2 and 8C). Therefore, the U-Pb age data from both sandstones and mélanges indicate the existence and later destruction of the middle-late-Cambrian intra-oceanic arc of the Junggar-Balkhash Ocean [42,83]. The Ordovician arc has better survived in the Tekturmas OB compared to the Itmurundy OB (Figs 3 and 4).

No serpentinite mélange has been reported in the Zharma OB of Kazakhstan, but several occurrences of ophiolitic mélange have been found in the Chinese part of the belt [16,42]. The early Carboniferous ages of zircons from Char sandstones match the coeval arc volcanics [58], implying that the intra-oceanic arc of that age has been relatively well preserved in the Char zone. The late-Devonian U-Pb ages of zircons from Char sandstones match those from Char igneous rocks, which either form small coherent bodies (Fig. 8B) or occur as fragments in serpentinite mélange [59] (Fig. 8D and E). Therefore, a late-Devonian to early-Carboniferous arc once existed in the Ob-Zaisan Ocean, but was tectonically eroded in late Carboniferous time.

Figure 9 presents a scheme of Pacific-type convergent margins evolving through both periods of accretion (Fig. 9A and C) and subduction erosion (Fig. 9B and D). At Stage A, at different times, there existed the Itmurundy-Tekturmas middle-late Cambrian arc (central Kazakhstan) and the Char late-Devonian arc (eastern Kazakhstan). At Stage B, those arcs were almost completely destroyed and serpentinite mélanges with fragments of older arcs were emplaced. At Stage C, an accretionary prism (early Ordovician in central Kazakhstan and early Carboniferous in eastern Kazakhstan) grew oceanward, and new arcs were initiated, and driven by continuing subduction the serpentinite mélanges were exhumed. At Stage D, the younger arcs were also partly eroded leaving thick turbidites and small survived arc terranes on the surface.

### Magmatic lull

**Figure 10.**
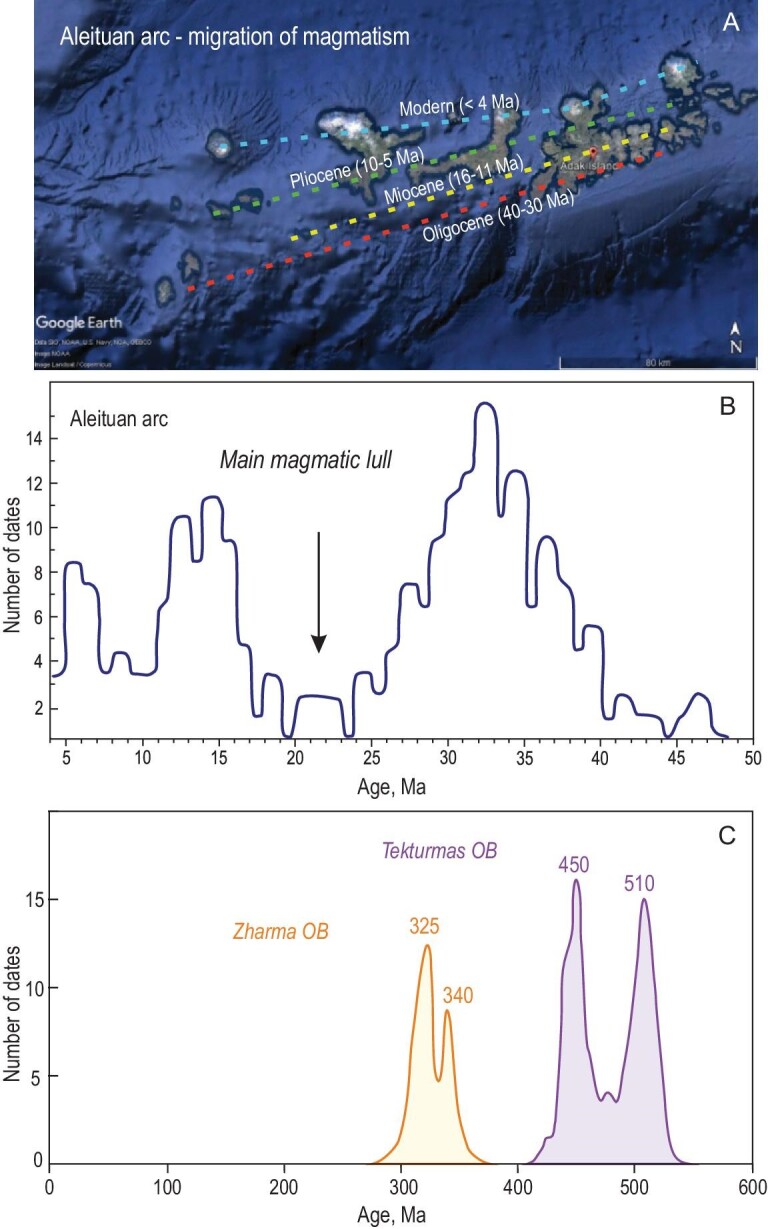
The magmatic lulls caused by the landward migration of the arc which is further related to subduction erosion. (A) A Google Earth image of the Aleutian arc with axes of magmatism younging in a direction opposite to subduction. (B) A summary of geochronological data for the Aleutian arc (adapted from Ref. [15]). (C) The U-Pb age distribution curves for zircons from greywacke sandstones of the Tekturmas and Zharma OBs showing magmatic lull between 500 and 460 and between 345 and 350 Ma, respectively.

Subduction erosion driven by the destruction of hanging wall (Fig. 1C and D) results in its subsidence and the shift of trench axis and magmatic arc landward (Fig. 9B and D). Magmatism typically attenuates and restores over a new subduction zone. Evidence for magmatic lulls caused by subduction erosion comes from age records of igneous rocks or from the distribution curves of U-Pb ages of zircons from arc-derived greywacke sandstones. In the case of young intra-oceanic arcs, we can directly measure the age of rocks. Such measurements made for the active Aleutian arc [15] showed that subduction erosion resulted in the landward migration of the axis of volcanism from 32 to 0 Ma (Fig. 10A) and also resulted in the magmatic lull at 24–17 Ma (Fig. 10B).

Our results from Kazakhstan show the magmatic lulls at 500–460 and 345–335 Ma, which are recorded in the U-Pb age distribution curves from the Yermek Fm. of the Tekturmas OB (Fig. 10C) and from the Koyandin Fm. of the Zharma OB (Fig. 10D), respectively. These magmatic lulls maintain the periods of subduction erosion in early Ordovician and early Carboniferous times at Pacific-type convergent margins of the Paleo-Asian Ocean.

## CONCLUSIONS

In order to reconstruct ancient Pacific-type convergent margins, we must know which types of arcs existed at that time: intra-oceanic or continental. Fossil Pacific-type orogenic belts typically exhibit very complicated relationships between different lithologies, often with few, if any, outcrops of arc igneous rocks. In this paper we reconstructed survived and disappeared Paleozoic intra-oceanic arcs of the Paleo-Asian Ocean based on published and new U-Pb detrital zircon ages, and petrographic, geochemial and isotope (Sm-Nd, Lu-Hf) data from greywacke sandstones hosted by accretionary complexes of central and eastern Kazakhstan, in comparison with data from arc igneous rocks, in particular those occurring as fragments in serpentinite mélange.

All sandstones are greywackes, of which the mafic to andesitic igneous protoliths were formed from juvenile mantle sources and were emplaced in an intra-oceanic arc setting. The sandstones were derived through destruction of middle-late-Cambrian and Ordovician arcs (Itmurundy and Tekturmas belts) and late-Devonian and Carboniferous arcs (Zharma and Char belts) and deposited in fore-arc/trench basins or, to a lesser degree, in back-arc basins.

The obtained results clearly show signatures of subduction erosion in both early and late Paleozoic times. Evidence for this comes from: (i) the disappearance of certain peaks of U-Pb ages in younger sandstones compared to older ones (Tekturmas, Char, Zharma); (ii) scarce/small outcrops of arc igneous complexes (Itmurundy, Char); (iii) the presence of pieces of arc rocks in serpentinite mélange (Itmurundy, Tekturmas, Char); (iv) magmatic lulls. The middle-late-Cambrian arcs (Itmurundy, Tekturmas) were fully destroyed by subduction erosion. Some of the Ordovician arc survived, but the arc of the Itmurundy belt was destroyed more comprehensively than the coeval arc of the Tekturmas belt. More of the late Devonian arc survived in the Zharma belt than did that in the Char belt. Both the early and late Paleozoic active margins of the PAO were characterized by alternating periods of accretionary growth and subduction erosion.

## Supplementary Material

nwac215_Supplemental_File
